# The Marine Spatial Planning Index: a tool to guide and assess marine spatial planning

**DOI:** 10.1038/s44183-023-00022-w

**Published:** 2023-09-26

**Authors:** Julie M. Reimer, Rodolphe Devillers, Rachel Zuercher, Pascale Groulx, Natalie C. Ban, Joachim Claudet

**Affiliations:** 1 Department of Geography, Memorial University of Newfoundland and Labrador, St. John’s, NL Canada; 2 Marine Planning & Conservation, Fisheries and Oceans Canada, Ottawa, ON Canada; 3 Espace-Dev (UMR 228), Institut de Recherche pour le Développement (IRD), Université de la Réunion, SEAS-OI, Saint-Pierre, La Réunion France; 4 University of Rhode Island, Narragansett, RI USA; 5 Biodiversity and Ecosystem Science, Fisheries and Oceans Canada, Ottawa, ON Canada; 6 School of Environmental Studies, University of Victoria, Victoria, BC Canada; 7 National Center for Scientific Research, PSL Université Paris, CRIOBE, CNRS-EPHE-UPVD, Maison de l’Océan, Paris, France

**Keywords:** Geography, Ocean sciences, Sustainability

## Abstract

Marine spatial planning (MSP) has the potential to balance demands for ocean space with environmental protection and is increasingly considered crucial for achieving global ocean goals. In theory, MSP should adhere to six principles, being: (1) ecosystem-based, (2) integrated, (3) place-based, (4) adaptive, (5) strategic, and (6) participatory. Despite nearly two decades of practice, MSP continues to face critical challenges to fully realize these principles, hindering its ability to deliver positive outcomes for people and nature. Here, we present the MSP Index, a tool for assessing progress in MSP processes based on MSP principles that can guide practitioners in operationalizing these principles. Using qualitative analysis of fundamental MSP guides, complemented with a literature review, we identified key features of MSP principles and developed these features into a scoring guide that assesses progress relative to each principle. We trialed and validated the MSP Index on six case studies from distinct regions. We found that the MSP Index allows for high-level comparison across diverse marine spatial plans, highlighting the extent to which MSP principles have permeated practice. Our results reveal successes, especially for the place-based principle, and failures to fully adhere to the adaptive and participatory principles of MSP. The Index serves as a guidance tool that would be best employed by practitioners and can inform science on the evolution of MSP. It is a user-friendly tool that translates MSP principles into practice, allowing for assessment of individual initiatives and comparison of diverse initiatives across ocean regions and nations.

## Introduction

Over the last 50 years, ocean-based industries have expanded at an increasing pace, representing a global acceleration in ocean development that is changing the ocean as it unfolds^1^. In addition to resulting in the rapid alterations of ecosystems, such rapid change may represent a loss to humanity of natural resources and other ecosystem services^2^. As nations develop aspirations for a sustainable blue economy – a pathway for bridging economic development with ocean stewardship, protection, and restoration^3–7^ – the need for coordinated, collaborative, and comprehensive ocean planning becomes increasingly urgent^8–11^.

Marine spatial planning (MSP) is a process for analyzing and informing the spatial and temporal distributions of ocean uses to achieve ecological, economic, and social objectives^12^. It offers a more holistic approach than traditional single-sector planning by accounting for multiple uses and objectives, while adopting some concepts from terrestrial planning^13,14^. MSP can help coordinate and regulate the blue economy by identifying sites for new ocean uses and compatible uses (e.g., fisheries and tourism), mitigating conflicts, enabling adaptation to changing conditions and priorities, fostering collaboration, and promoting capacity building^8^, while ensuring that efforts to realize the economic potential of the ocean do not damage already fragile ecosystems. At its core, MSP strives to achieve balance, holding the potential to deliver both ocean conservation and sustainable use or development objectives^15,16^. There is a strong and growing body of academic research and theory behind MSP^17,18^, but MSP will not fulfill its potential for supporting global goals for a healthy and productive ocean if this theory cannot be translated into practice^19^.

In their influential step-by-step guide to MSP, Ehler & Douvere^12^ identified characteristics of effective MSP: (1) ecosystem-based, (2) integrated, (3) place-based or area-based (hereafter, place-based), (4) adaptive, (5) strategic and anticipatory (hereafter, strategic), and (6) participatory. Here, we consider these characteristics to be foundational principles of MSP, aligning with those guiding MSP in the European Union (EU). For instance, the EU principal for “using MSP according to the area and type of activity” mirrors the place-based principle; “incorporating monitoring and evaluation” reflects the adaptive principle; and “coordination with Member States” aligns with the integrated principle^20^. The practical application of these principles has proven challenging, as many MSP initiatives struggle to varying extents to effectively adapt plans, engage stakeholders, strengthen institutions, and/or balance economic development with conservation^15,21^. MSP initiatives are diverse^19^, and often driven by political interests and investments^22^, resulting in plans that unevenly employ best practices and may or may not support a sustainable blue economy.

As many initiatives worldwide are in pre-planning and plan preparation phases of MSP^21^, and given the growing prominence of blue economy discourses and policies^23,24^, now is a critical time for providing guidance that ensures MSP theory informs practice. Using qualitative analysis of fundamental MSP guides, complemented with a literature review, we identified key features of MSP principles and developed these features into a user-friendly tool that can assess progress in diverse MSP initiatives relative to these principles and best practices.

## Results

### The MSP Index

**Fig. 1:**
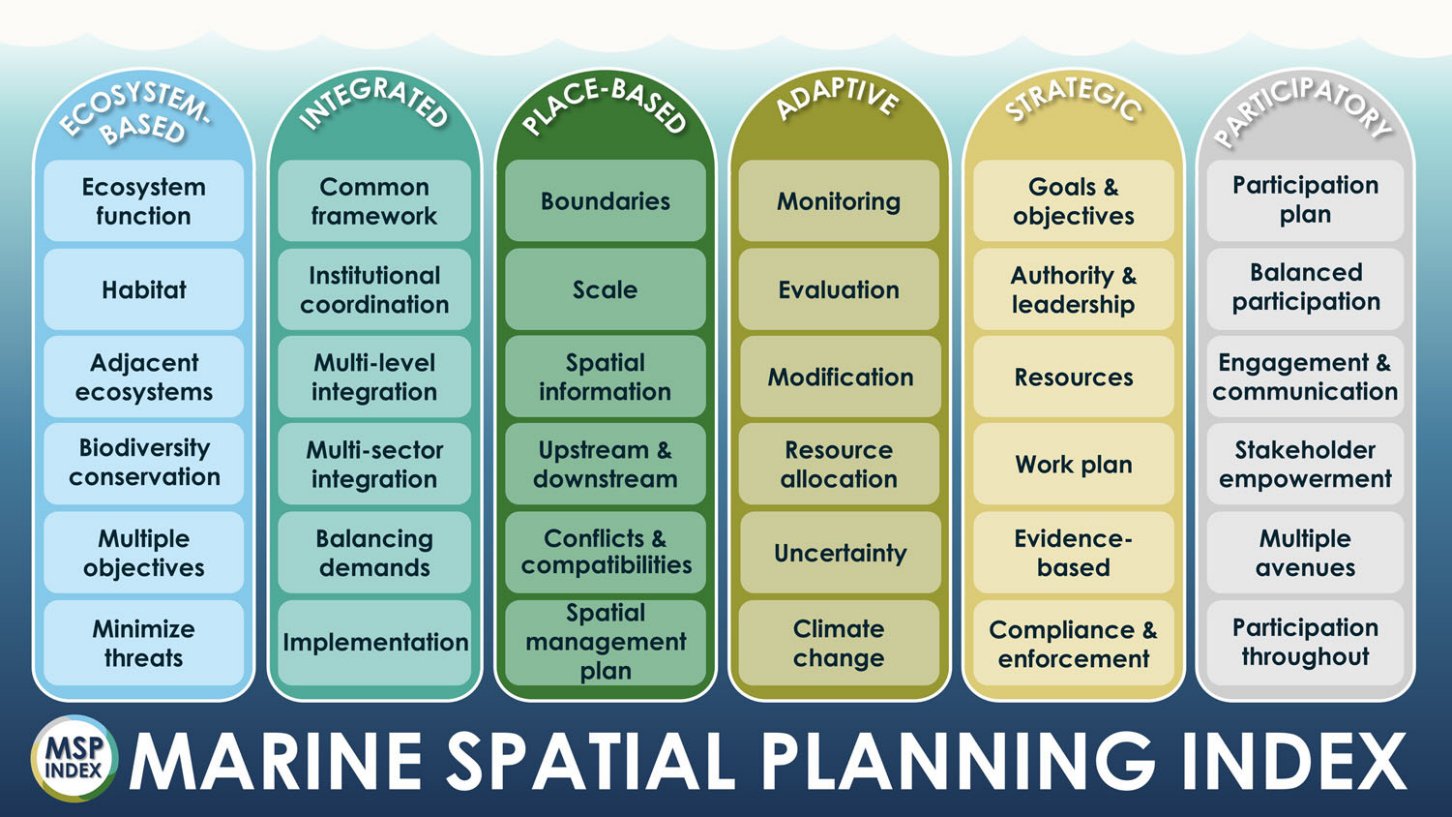
Features of the MSP Index under foundational principles ecosystem-based, integrated, place-based, adaptive, strategic, and participatory. To assess MSP progress, each feature can score between zero and three points based on feature criteria statements defined in the MSP Index scoring guide (Supplementary Table 2 and Fig. 2).

**Fig. 2:**
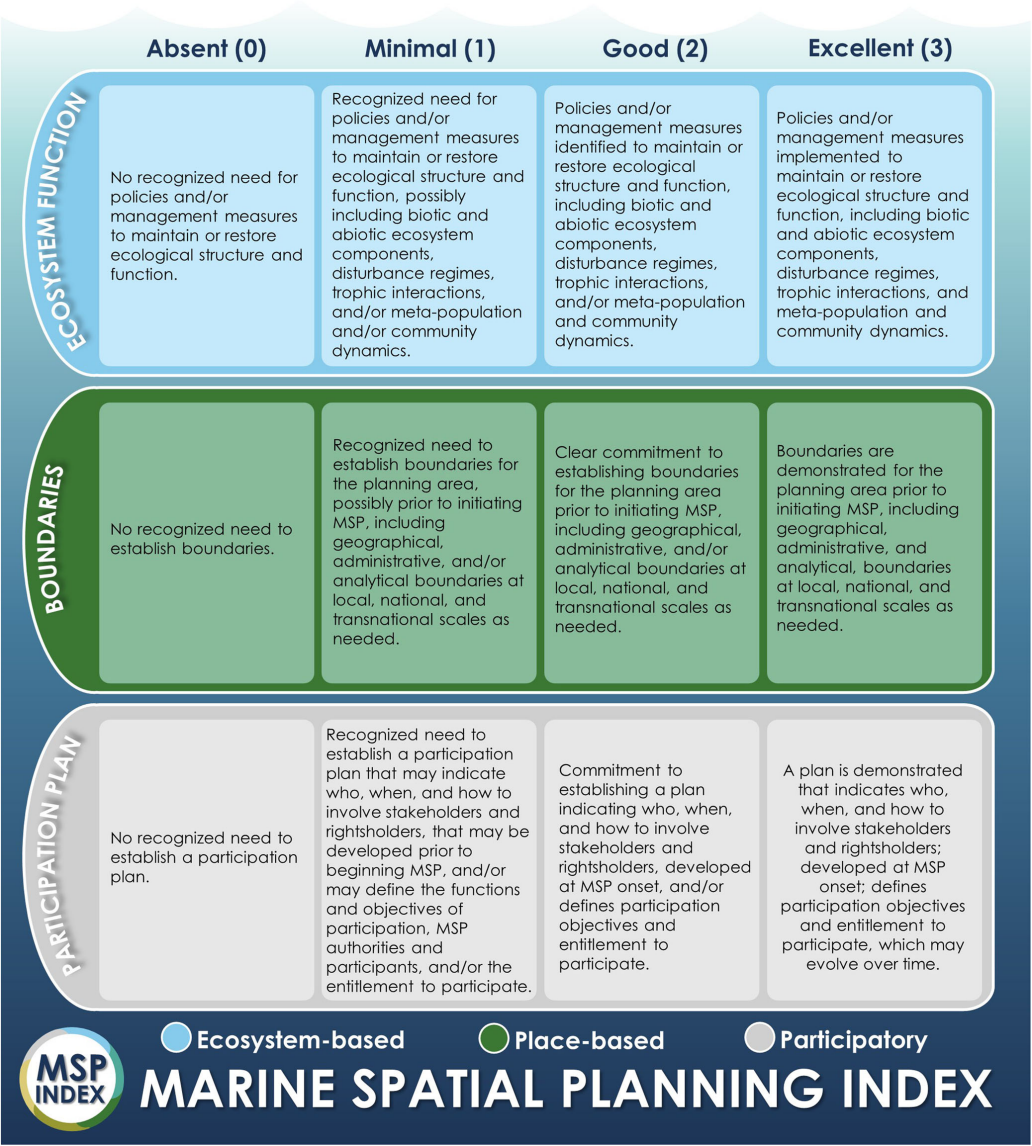
Example scoring guide for three features (out of 36) from the MSP Index in the ecosystem-based, place-based, and participatory principles (three principles out of six). Case studies were scored according to this guide (Supplementary Table 2 and MSP Index file provided in Supplementary Information).

The MSP index comprises 36 features ranging from establishing a common framework for integration in MSP, to monitoring, to setting goals and objectives (Fig. 1). Of the 36 features, 33 were identified, in some part, from Ehler & Douvere^12^ and Ehler^25^. Only climate change (adaptive), multi-level integration (integrated), and scale (place-based) emerged from the supplementary literature alone. Most features emerged from more than one source, though resource allocation, climate change, upstream and downstream, and spatial information emerged from single sources (i.e., one reviewed document). The features that comprise the Index broadly reflect best practices and core elements of MSP, providing a framework for assessing a plan or planning process as it relates to foundational principles. The features do not reflect MSP outcomes or relate to particular objectives (e.g., sustainable fishing practices, suitable areas for renewable energy development), nor does the Index aim to assess MSP outcomes or the efficacy of particular features (e.g., whether existing mechanisms for incorporating climate change result in effective climate adaptation or mitigation). Still, we believe that positive outcomes for both people and nature are more likely to be met when an MSP Index score is high. Criteria statements for features ranged from a lack of recognition or intention to achieve a feature to implementation of a feature, where requirements have been met (Fig. 2).

### Case study context

Context is important in MSP initiatives and influences the principles and key features emphasized in resulting marine spatial plans. Document analysis highlighted diversity among the analyzed case studies and their contexts, resulting in plans that differed in their goals, processes, and expected capacity to affect and implement policy and regulations. Most case studies had goals related to sustainability, including the sustainable use of natural resources, sustainable ecosystems, and the sustainable development of new ocean uses; however, the Kiribati case, while listed by the IOC as an MSP initiative, was distinct from the others in its strong focus on ecosystems, its closer alignment with marine protected area planning, and that it operates in a remote and largely unpopulated region. Because this case study was listed as an MSP initiative by the IOC, we did not exclude it from analysis. All case studies were led or adopted by government authorities, except for the Israel plan that was primarily developed by a team of academic researchers, planners, and consultants. In this case, governments were stakeholders who participated in the MSP process.

While some plans established an MSP policy framework, others focused on regulations and zoning. The Bataan initiative was the only case study to establish zones for all uses and objectives (e.g., aquaculture zone, municipal fishing zone, sanctuaries). The Rhode Island case established zones for only renewable energy development. The Rhode Island case was also the only initiative analyzed that established regulations, though these regulations were also specific to minimizing the impact of renewable energy developments on existing uses and the ecosystem. This plan occurred at the state-level with linkages to national-level policy and legislation. In contrast, the Ireland and South Africa case studies are national-level initiatives that established frameworks for decision-making concerning marine uses and planning.

### Trialing the MSP Index

**Fig. 3:**
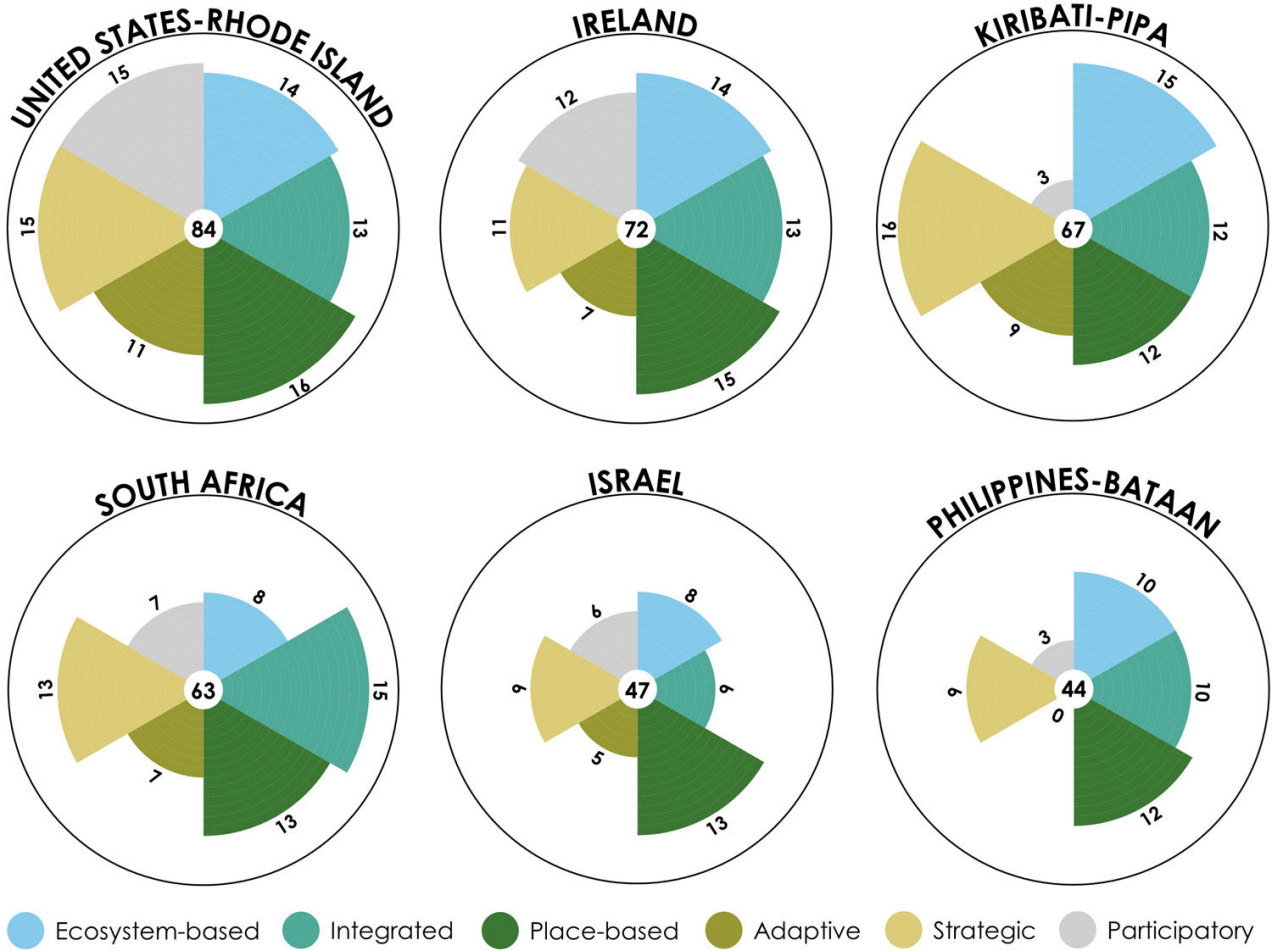
MSP Index scores for assessed case studies. Each petal represents the score per MSP principle (ecosystem-based, integrated, place-based, adaptive, strategic, and participatory). The score per principle is depicted by the number at the outer edge of each petal, with a maximum possible score per principle of 18. The overall MSP Index score is depicted by the number in the centre of each flower plot, with a maximum possible overall MSP Index score of 108.

We found that the MSP Index was flexible enough to be applied to the diverse case studies selected (Table 1), providing a high-level snapshot of progress made toward realizing MSP principles in each initiative (Fig. 3). Of a possible maximum 108 points, the initiatives scored between 44 points (*Coastal Land- and Sea-use Zoning Plan of the Province of Bataan)* and 84 (*Rhode Island Ocean Special Area Management Plan*). On average, the place-based principle scored highest across plans (13.5 out of 18 possible points). The lowest average scores were found for the adaptive (6.5 out of 18 possible points) and participatory principles (7.7 out of 18 possible points). For the remaining principles, average scores were 11.5 (ecosystem-based), 11.5 (integrated), and 12.2 (strategic) out of 18 possible points. The highest score for any principle was 16, achieved by the Rhode Island case for the place-based principle and the Kiribati case for the strategic principle. For all case studies, only nine of 36 principles (six per case study) scored 14 or higher, and four of these instances belonged to the Rhode Island initiative.

**Table 1:** Characteristics of case studies selected to trial functionality of the MSP index.

Case study	Year	Scale	Intention
Ireland	2021	490,000 km^2^	Marine planning
Israel	2015	26,000 km^2^	Marine planning
Kiribati – PIPA*	2015	408,250 km^2^	Conservation planning
Philippines – Bataan	2007	Up to 15 km municipal limit	Coastal zone planning
South Africa	2017	472,280 km^2^	Marine planning
USA – Rhode Island	2010**	3800 km^2^	Marine planning

Intention reflects the high-level purpose of each case study, where coastal zone planning focuses on integrated planning in that zone, conservation planning focuses on the protection of biodiversity and ecosystems, and marine planning encompasses a form of MSP with a broader set of goals.

*PIPA Phoenix Islands Protected Area.

**Revisions of general policies and regulatory standards adopted January 10, 2012.

Within principles, features were most often scored as good (score = 2; 43% of scores), followed by minimal (score = 1; 23% of scores), excellent (score = 3; 22% of scores), and absent (score = 0; 13% of scores). Resource allocation (adaptive) scored as absent for all case studies, while compliance and enforcement (strategic) and balancing demands (integrated) scored as minimal for all but Rhode Island and Kiribati cases. Uncertainty (adaptive) also scored as minimal for all but the Rhode Island case study. Under the participatory principle, stakeholder empowerment and participation plan both scored as absent for half of the case studies. In contrast, boundaries (place-based) scored as excellent for all cases except Israel, and spatial information (place-based) scored as excellent for all but the Kiribati and Bataan case studies (Supplementary Data).

Within case studies, the Rhode Island case scored above average for all MSP principles, while the Israel and Bataan cases scored below average for all principles. The Israel case scored 50% of the maximum possible principle score (18) or less for all principles except place-based. The Bataan initiative was the only case study to score zero on a principle (adaptive). The Kiribati (*Phoenix Islands Protected Area Management Plan 2015-2020*) and Bataan case studies scored below average for the participatory principle.

## Discussion

We developed the MSP Index to allow for a high-level assessment of marine spatial plans that can inform on their comprehensive nature and point to potential areas of improvement. The majority of features included in the Index were widely supported by the literature, and are well-aligned with recognized enabling conditions for effective MSP^26^. The MSP Index gives a snapshot of the extent to which theoretical principles have permeated MSP practice. With this snapshot, the MSP Index can highlight successes and gaps in MSP initiatives. It may also support practitioners in grounding MSP in best practices at early stages of the process or in identifying areas for improvement, areas for capacity or resource investment, strategic prioritization of key features based on management needs, and potential operational risks as the process evolves.

We found that the Index can be used to compare different types of MSP initiatives, from local to national-scale plans, recent and older plans, and plans with diverse objectives in sustainable resource use and biodiversity conservation. The case studies analyzed to test functionality of the Index revealed that while some principles are clearly intrinsic to the MSP process, like place-based that consistently scored high among analyzed initiatives, others appear more challenging to implement. We found that only 25% of MSP principles scored 14 or higher across case studies (maximum score = 18), resonating with persistent challenges facing MSP development, including deficiencies in political and institutional frameworks; stakeholder engagement; balancing economic development with conservation, and incorporating global environmental change^15^. These challenges hinder the use of MSP principles in practice, reflected here under the integrated, participatory, ecosystem-based, and adaptive principles, respectively. Our case study analysis generally shows how MSP principles have been unevenly applied in practice. As MSP processes often exist within complex and well-established governance systems, the application of MSP principles in practice may be affected by the norms, procedures, and limitations of such systems. The variation in principle scores across the analyzed case studies may indicate possible trade-offs between employing all MSP principles in complex processes to address broad challenges, resulting in plans that may be difficult to implement, monitor, and evaluate, and selectively employing principles and their key features in less complex processes to address specific challenges, resulting in plans that may be more feasible to implement.

We found that the adaptive principle scored lowest across analyzed case studies, suggesting that plans are seen as an end instead of a continuing process; however, MSP is not intended to result in a definitive plan, but should be approached like other planning processes, such as urban planning, which are iterative to ensure that the plan remains relevant^12^. Once plans are complete, the incentive for governments to continue investment in MSP likely diminishes. There are few clear examples of MSP initiatives that embrace change, dynamic systems, and adaptation^27,28^, and initiatives seldom dedicate sufficient resources to monitoring, evaluation, and adaption^15^. These challenges were especially apparent in our analysis, as four case studies scored minimal or absent for the evaluation feature and no case study met the criteria for resource allocation under the adaptive principle (though it is possible that these features exist in practice and have not been publicly documented). Without explicit attention to and resources for evaluation, it is difficult to disentangle the actual outcomes of MSP from outcomes related to all other elements affecting ocean activities and ecosystems^29^. This challenge is also reflected at a global scale, where evaluation in MSP has shifted away from evaluation of outcomes to evaluation of the MSP process itself^30^. A similar trend has been observed in conservation, where political and institutional barriers to assessing conservation impacts can be pervasive and difficult to overcome^31,32^. To demonstrate adherence to the resource allocation feature under the adaptive principle, a plan should have mechanisms to allow reallocation of resources away from ineffective management actions to alternatives identified through monitoring and evaluation (Supplementary Table 2). No case studies met this criterion, suggesting that the analyzed plans may be under-prepared for iterative planning and adaptation.

Similarly, though to a lesser extent, we showed using the MSP Index that many initiatives lack key features of a participatory process. Recent MSP initiatives appear devoid of politics^22^, despite MSP being an intrinsically political process^12^. This unpolitical version of MSP sanitizes the process toward consensus, likely disempowering stakeholders with diverse and contrasting views^22^. Through the MSP Index, our analysis may confirm this, as the stakeholder empowerment feature scored consistently low. To achieve an excellent score for this feature, an initiative must demonstrate that mechanisms exist to ensure stakeholders have the means, skills, and knowledge to participate in MSP, among other criteria (Supplementary Table 2). Others have found experiences among MSP participants that the process is exclusionary, plagued by poor communication, fragmented governance, and vagueness surrounding winners and losers in MSP^33,34^. To be properly participatory, MSP initiatives must distinguish between inviting stakeholders to the table and empowering them to influence MSP outcomes, including policy^35^.

The MSP Index is intended to give a high-level overview of MSP initiatives in development and implementation stages, but it does not evaluate the efficacy of MSP against objectives, the efficacy of individual key features, nor does it fully discern the intention or context of plans. While the MSP Index can assess whether mechanisms exist to ensure stakeholders are empowered to participate in the process, it does not assess whether such mechanisms are effective or meaningful. Even so, at a high-level, the MSP Index can indicate the extent to which key features have been advanced. We believe that a high MSP Index score reflects an MSP initiative that is more likely to deliver ecological, economic, and social objectives as intended. To maximize utility of the MSP Index in varied contexts, we recommend that Index scores be accompanied by a description of the analyzed plans to reflect local realities and challenges that influence whether, how, and when MSP principles are implemented. For example, the Israel case study scored below average for all principles and scored lowest of all case studies on the integrated principle; however, this plan was neither led nor adopted by government authorities. While the plan often recognizes the need for mechanisms to achieve key features of the MSP Index (e.g., institutional coordination, multi-level integration, balancing demands), it lacks the authority to commit to or implement these mechanisms. The Kiribati case study scored low on the participatory principle; however, the Phoenix Island Protected Area (PIPA) region lacks permanent human settlement and, at the time of this plan, was inhabited by fewer than 40 people employed as government caretakers for the protected area^36^. Given this, the participatory principle may not be as applicable to this case study as for others assessed here due to a lack of local users. In such cases, practitioners applying the Index might omit or adapt principles and features to suit local needs and MSP objectives. Over time, principles may become more or less relevant to an MSP initiative. In the case of PIPA, as the area is opened to commercial fishing for the first time since 2015^37^, a participatory and inclusive process may be necessary for future iterations of MSP.

Despite recent growing recognition of the importance of culture for ocean planning and management^38–40^, cultural values have not been widely embraced in MSP^41^. As presented here, the MSP Index lacks a direct cultural component, which may reflect the lower relative importance given to culture when fundamental MSP guides were published. Still, cultural aspects important to MSP are captured by some features in the MSP Index. For example, criteria for the evidence-based feature includes use of the different types of information, such as Indigenous and local knowledge; criteria for the stakeholder empowerment feature includes decentralizing management or enabling participation in governance; criteria for the common framework feature requires that such frameworks integrate within and between Rightsholders, stakeholders, governance, policy, legislation, and management; and criteria for the balancing demands feature includes evaluating trade-offs among ecological, social, cultural, and economic objectives and activities (Supplementary Table 2). These criteria may be extracted from existing features and added to a future iteration of the Index that more directly incorporates culture. A culture-related MSP principle may include features such as dedicated funds for collecting sociocultural data, investment in reliable partnership building and knowledge co-production, co-management, or commitments to equitable decision-making and outcomes^42,43^.

The case study analyses we present are limited by the realities of external review, including access to only publicly available documents, which likely do not capture MSP initiatives in their entirety. Our application of the MSP Index focused on final marine spatial plans, and was supplemented with relevant webpages, legislation, and documents as necessary. Still, this method is limited to documents that are freely available, and it is likely that files in progress or sensitive in nature, including those pertaining to the adaptive and participatory principles, are not made available to the general public. Further, we did not assess all complementary management plans or policies that may contribute to comprehensive MSP (e.g., management plans of marine protected areas referenced in final marine spatial plans). Given this, it was difficult to discern some features. For example, if an initiative is further along in the MSP process, a work plan may exist but may not be reported in the current iteration of the plan. For a feature to score ‘excellent’ (3), all requirements of said feature must be clearly present in the analyzed documents. This may have contributed to nearly twice as many features scoring ‘good’ (2), rather than ‘excellent’ across case studies. The Rhode Island case develops a strong spatial management plan, but it is not clear from the plan alone whether a preferred scenario was selected from alternatives. Since the plan did not meet all requirements of this feature, it was scored as ‘good’. Future applications of the Index by external reviewers may couple document analysis with practitioner interviews. Secondly, future iterations of the Index may be more flexible if an excellent score required the majority of requirements to be present, rather than all. In general, the MSP Index would be best used by MSP practitioners and case study experts who are aware of the complete context of assessed initiatives beyond what is published in publicly available documents.

The MSP Index proved to be a flexible tool for assessing MSP processes based on foundational principles of being ecosystem-based, integrated, place-based, adaptive, strategic, and participatory. The Index uses a qualitative scoring guide to assess key features under these principles that reflect MSP best practices, highlighting successes and gaps in MSP processes, such as areas for capacity or resource investment, operational risks, and systemic barriers to MSP advancement, to inform a path forward. Since many MSP initiatives and resulting marine spatial plans are developed over the span of several years^8^, the Index may support MSP process evaluation through multiple applications over time, demonstrating progress within an initiative as it moves toward best practices across MSP principles. Our application of the Index to six case studies reveals that MSP principles are unevenly applied in practice, which may reflect the diversity of approaches to, objectives for, and localized needs of MSP. While the Index is based on best practices derived from fundamental MSP guides^12,25^, we designed it to be flexible to adaptation; future iterations might incorporate new principles or features that are locally relevant. This may include a cultural component, given the need to incorporate cultural considerations in governance for effective and equitable ocean management and sustainability^38,44,45^. The MSP Index is a user-friendly tool to gauge progress based on MSP principles, allowing for assessment of individual MSP processes as they evolve and comparison of diverse initiatives around the world.

## Methods

### Identifying MSP features

**Fig. 4:**
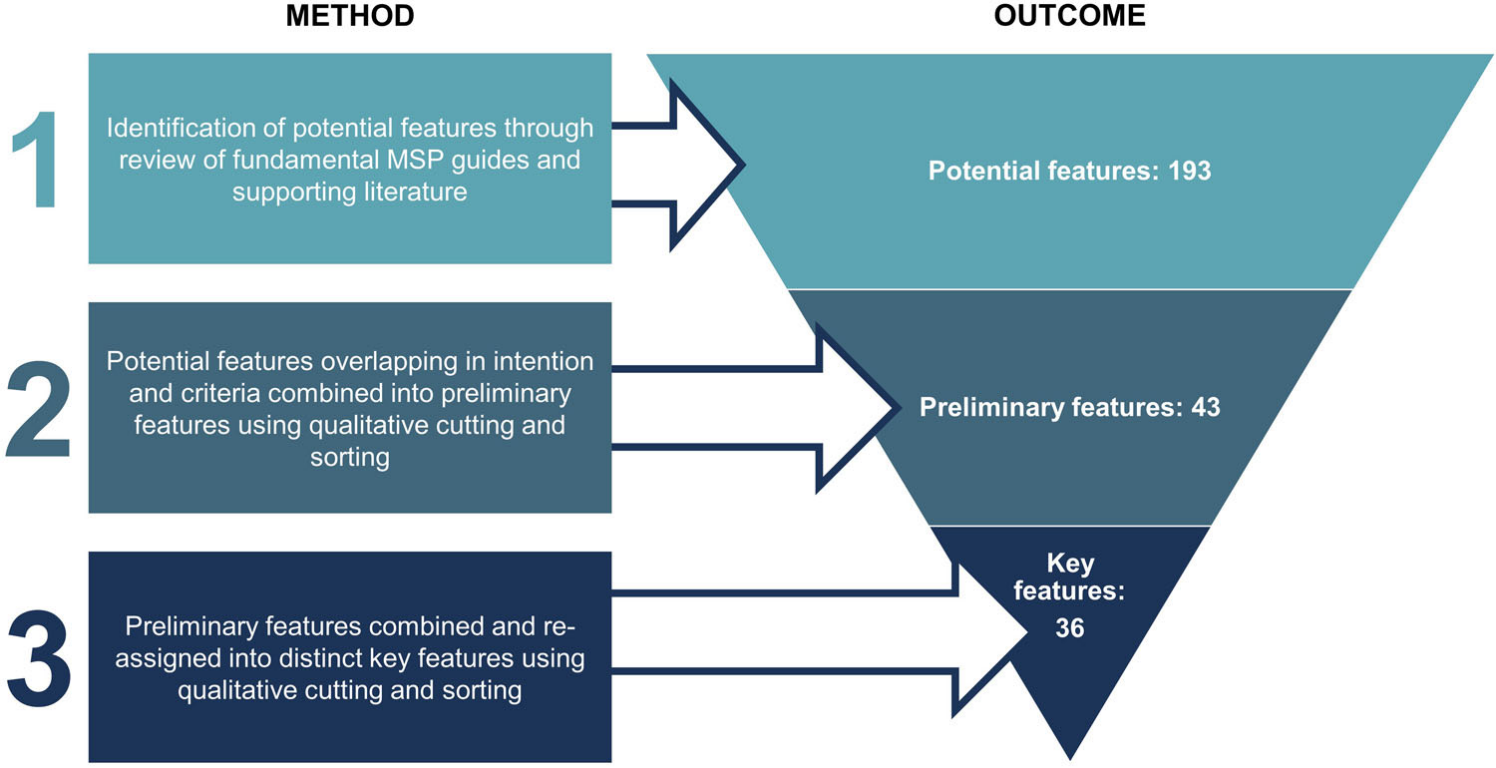
Three-step method for identifying potential, preliminary and key features of the MSP Index. Outcomes from each step of this method are shown. The initial 193 potential features identified through review of key literature underwent two rounds of qualitative cutting and sorting to establish the final 36 key features.

We used a three-step process to identify and describe key MSP features of the six MSP principles. We define key features as distinct attributes of MSP principles that, when implemented, ensure principles are present in MSP (e.g., biodiversity conservation is a feature of the ecosystem-based principle). Key features are defined by a set of criteria to be effectively implemented (e.g., to effectively implement biodiversity conservation, management measures must exist to maintain or restore biodiversity, their habitats, and ecological processes). The three-step process involved (1) a literature review and qualitative document analysis to identify potential features; (2) qualitative sorting to identify preliminary features; and (3) qualitative sorting to amalgamate and describe key features (Fig. 4). First, potential features were derived from a review of fundamental MSP guides, including Ehler & Douvere’s^12^ step-by-step guide and Ehler’s^25^ guide to evaluating marine spatial plans. At the time of review, the recent international MSP guide^8^ had not yet been published. This review was supplemented with select papers that are widely accepted as leading publications about MSP based on the number of citations or publications authored by subject matter experts (expertise determined by the number of articles on a topic by the author(s)^46^) (Supplementary Table 1). Our intention was to develop an index that could be flexible enough to be adapted with alternative features as needed by MSP practitioners in response to the unique realities of planning areas. Given this, it was deemed unnecessary to conduct a systematic literature review to identify all possible features under MSP principles, though we are confident that MSP best practices have been captured.

For document analysis, we used a blended approach to qualitative coding to identify features from the MSP guides and selected supplementary literature^47^. Passages of text were deductively assigned a code for the potential principle they reflected (e.g., adaptive or participatory) and inductively assigned a code for a potential feature (e.g., uncertainty or stakeholder dialogue) as they emerged from the text. Analysis of the selected literature resulted in 193 potential features. Potential features overlapped in their intention or, in some cases, better reflected potential requirements (i.e., descriptive elements or specific actions to be taken to fulfill a feature). We used cutting and sorting of the coded passages of text to group similar items together^48^, establishing a set of 43 preliminary features. For each of these, we described an intention and retained potential requirements of features identified from the coded passages of text. Following this, we used a second round of cutting and sorting to amalgamate preliminary features where there were redundancies and to ensure best fit of the features to their respective MSP principles (Fig. 4 and Supplementary Fig. 1). This process resulted in a set of 36 key features, six per MSP principle, each with distinct intentions and requirements.

### Developing & trialing the MSP Index

Using the identified features, we developed the MSP Index – a qualitative scoring guide that can be used to assess progress in MSP processes as it relates to MSP principles. In this guide, we used a four-point scale, from zero to three points. A zero measure indicates the absence of a feature from a given plan, while one to three points capture the varying extents to which a plan or MSP process meets feature criteria. For each possible score, we developed a concise criteria statement based on intentions and requirements of the feature. In our Index, a feature can be *absent* (score = 0); *minimal*, where a feature is generally present, but few requirements are present (score = 1); *good*, where commitments to a feature are made, but not all requirements are present (score = 2); or *excellent*, where all requirements are clearly present in an MSP initiative (score = 3). Criteria for *good* scores generally use “and/or” statements for requirements, while criteria for *excellent* scores use more definitive and exclusive “and” statements. To ensure consistency in scoring, in the event that most but not all requirements of a feature are present, that feature is always scored as *good*. An MSP initiative in-and-of-itself need not be responsible for the advancement of a feature for it to be assessed by the MSP Index.

To trial the functionality of the MSP Index, we applied the scoring guide to six international case studies selected from the MSP online database of the Intergovernmental Oceanographic Commission (IOC) (http://msp.ioc-unesco.org, accessed in June 2021). It should be noted that, as of January 2023, content from this website has been migrated to MSPglobal, an initiative of the IOC (http://www.mspglobal2030.org/msp-around-the-world/). We provide a list of the MSP initiatives in the Supplementary Data. To capture a diversity of MSP processes and ensure representativity, we used stratified random sampling to identify one case study for each of the six regions identified in the database: Africa (*n* = 10 MSP initiatives), Asia (*n* = 8), Europe (*n* = 38), Middle East (*n* = 2), Oceania (*n* = 10), and the Americas (*n* = 38). Each of the 106 MSP initiatives was assigned an identifier number and all initiatives within a given region were arranged in numerical order. We used R Version 3.6.1 to randomly sample case studies by identifier number from each ocean region, then screened the associated case study using the following criteria:

- Language: the case study documentation must be in English due to language limitations of the lead author
- Plan: the case study must have a final draft or final approved plan available
- Supporting content: the case study must have sufficient content publicly available

If a randomly selected case study did not meet these criteria, then we continued the random sampling without replacement until a case study was selected that met the criteria. For most regions, the first or second case study screened met the inclusion criteria, except for Africa where only the sixth case study screened met the criteria, primarily due to a lack of publicly available documents. The six selected case studies capture MSP initiatives from different years of completion, at different scales, and with different intentions (Table 1).

We applied the MSP Index to these case studies using document analysis and qualitative coding, the process of labelling and organizing passages of text, in QSR International’s NVivo-12 software^47,49^. Final marine spatial plans were the primary documents used (*Coastal Land- and Sea-use Zoning Plan of the Province of Bataan*^50^*; National Framework for Marine Spatial Planning in South Africa*^51^*; Phoenix Islands Protected Area Management Plan 2015-2020*^52^*; Project Ireland 2040 National Marine Planning Framework*^53^*; Rhode Island Ocean Special Area Management Plan; The Israel Marine Plan*^54^), but in cases where scoring of a feature was unclear, we reviewed grey literature (including webpages, legislation, guiding documents and frameworks, participation documents, and government documents) for additional information. Passages of text within these documents were coded to features under the MSP principles. Once all documents had been coded, we reviewed the related passages of text to score each feature using the guide. Feature scores were then summed for each principle to determine a principle score (out of 18); all six principle scores were summed to determine the overall MSP score (out of 108).

### Supplementary information

Supplementary Information

Supplementary Data

## Data Availability

Data supporting the analyses and results of this study are available in the Supplementary Data. Correspondence regarding this data should be addressed to JMR.
