# Supplementary material for: The Marine Spatial Planning Index: a tool to guide and assess marine spatial planning
Source: NPJ Ocean Sustain. 2023 Sep 26;2(1):15. doi: 10.1038/s44183-023-00022-w (PMC11062297; doi:10.1038/s44183-023-00022-w)
Supplement: Supplementary file 1 — Supplementary Information [file 44183_2023_22_MOESM1_ESM.docx]

**The Marine Spatial Planning Index: a principle-based tool to guide and assess marine spatial planning**

Julie M Reimer, Rodolphe Devillers, Rachel Zuercher, Pascale Groulx, Joachim Claudet

**SUPPLEMENTARY INFORMATION**

**Table of Contents**

[Supplementary Figure 1 3](#_Toc144453218)

[Supplementary Table 1. 3](#_Toc144453219)

[Supplementary Table 2. 5](#_Toc144453220)

******Supplementary Figure 1.** Detailed identification of the final 36 key features in the MSP index, established through qualitative cutting and sorting of 43 preliminary features, for each MSP principle: (A) adaptive, (B) ecosystem-based, (C) integrated, (D) participatory, (E) place-based, and (F) strategic.

| **Supplementary Table 1.** Key literature used to identify features under each MSP characteristic. “Type” refers to either MSP-focused literature (“MSP”) or other relevant literature (“Other”). The MSP principle listed for each item is not necessarily exclusive, as many of these resulted in the identification of features for other principles following qualitative cutting and sorting. | | |
| --- | --- | --- |
| **MSP Principle(s)** | **Citation** | **Type** |
| All | Ehler, C. & F. Douvere. (2009). Marine Spatial Planning: a step-by-step approach toward ecosystem-based management. Intergovernmental Oceanographic Commission and Man and the Biosphere Programme, <http://msp.ioc-unesco.org/msp-guides/msp-step-by-step-approach/> | MSP |
| All | Ehler C. (2014). A Guide To Evaluating Marine Spatial Plans. IOC Manuals and Guides, http://msp.ioc-unesco.org/msp-guides/evaluating-marine-spatial-plans/ | MSP |
| Adaptive | Frazão Santos, C., Agardy, T., Andrade, F., Calado, H., Crowder, L.B., Ehler, C.N., García-Morales, S., . . . & R. Rosa. (2020). Integrating climate change in ocean planning. Nature Sustainability, <http://doi.org/10.1038/s41893-020-0513-x> | MSP |
| Ecosystem-based | Borgström, S., Bodin, O., Sandström, A. & B. Crona. (2015). Developing an analytical framework for assessing progress toward ecosystem-based management. *AMBIO, 44,* <http://link.springer.com/10.1007/s13280-015-0655-7> | Other |
| Ecosystem-based | Convention on Biological Diversity. (2010). Ecosystem approach principles, <https://www.cbd.int/ecosystem/principles.shtml> | Other |
| Ecosystem-based | Foley, M.M., Halpern, B.S., Micheli, F., Armsby, M.H. & M.R. Caldwell. (2010). Guiding ecological principles for marine spatial planning. *Marine Policy, 34,* <http://dx.doi.org/10.1016/j.marpol.2010.02.001> | MSP |
| Ecosystem-based | Leslie, H.M. & K.L McLeod. (2007). Confronting the challenges of implementing marine ecosystem-based management. Frontiers in *Ecology and the Environment, 5,* <http://doi.org/10.1890/060093> | Other |
| Ecosystem-based | UNEP. (2011). Taking Steps toward Marine and Coastal Ecosystem-Based Management - An Introductory Guide. UNEP Regional Seas Reports and Studies No. 189, https://wedocs.unep.org/handle/20.500.11822/8445 | Other |
| Integrated | Asif, M., Zutshi, A. & N. Ahmad. (2011). An integrated management systems approach to corporate sustainability. *European Business Review, 23,* <https://doi.org/10.1108/09555341111145744> | Other |
| Integrated | Stephenson, R.L., Hobday, A.J., Cvitanovic, C., Alexander, K.A., Begg, G.A., Bustamante, R.H., Dunstan, P.K., . . . T.M. Ward. (2019). A practical framework for implementing and evaluating integrated management of marine activities. *Ocean & Coastal Management, 177,* <http://doi.org/10.1016/j.ocecoaman.2019.04.008> | Other |
| Participatory | Kidd, S. & L. McGowan. (2013). Constructing a ladder of transnational partnership working in support of marine spatial planning: Thoughts from the Irish Sea. *Journal of Environmental Management, 126,* <http://doi.org/10.1016/j.jenvman.2013.03.025> | MSP |
| Participatory | Morf, A., Kull, M., Piwowarczyk, J. & K. Gee. (2019). Towards a Ladder of Marine/Maritime Spatial Planning Participation. In Zaucha, J. & K. Gee (Eds.) *Maritime Spatial Planning,* <http://link.springer.com/10.1007/978-3-319-98696-8_10> | MSP |
| Participatory | Twomey, S. & C. O’Mahony. (2019). Stakeholder Processes in Marine Spatial Planning: Ambitions and Realities from the European Atlantic Experience. In Zaucha, J. & K. Gee (Eds.) *Maritime Spatial Planning,* <http://link.springer.com/10.1007/978-3-319-98696-8_13> | MSP |

| **Supplementary Table 2.** The MSP index scoring guide, using a four-point scale from zero to three, maximum possible points is 108. Absent means there is no intention or recognition of a feature; minimal means there is an intention or recognition of a feature; good means there is a commitment to and identification of a feature, and more requirements of a feature are present, and excellent means the feature fully exists with all requirements. | | | | | |
| --- | --- | --- | --- | --- | --- |
| **Feature** | | **Absent (0 pts)** | **Minimal (1 pt)** | **Good (2 pts)** | **Excellent (3pts)** |
| **Adaptive** | Monitoring | No recognized need to monitor for management outcomes. | Recognized need to monitor, possibly through routine or systematic processes, for management outcomes. A baseline description of the initial state of the system may not exist and performance indicators may not be identified. | Clear commitments and/or one-time investments are made to allow routine and/or systematic monitoring for management outcomes, compared against a baseline description of the initial state of the system using qualitative and/or quantitative performance indicators. | Reliable investments are demonstrated to allow routine and systematic monitoring for management outcomes, compared against a baseline description of the initial state of the system using qualitative and/or quantitative performance indicators. |
|  | Resource allocation | No recognized need for resource reallocation to enable adaptation. | Recognized need for resource reallocation to enable adaptation. | Mechanisms identified to enable resource reallocation away from ineffective management actions, possibly based on monitoring and evaluation, to alternatives and/or to assess lower cost management alternatives for adaptation. | Mechanisms demonstrated to enable resource reallocation away from ineffective management actions, based on monitoring and evaluation, to alternatives and to assess lower cost management alternatives for adaptation. |
|  | Evaluation | No recognized need to establish an evaluation plan. | Recognized need to establish an evaluation plan for assessing efficacy and/or ability to achieve MSP objectives, possibly including procedures for regular analysis and interpretation of monitoring data to inform adaptation needs and/or for open and accessible reporting of evaluation findings. | Clear commitment to establishing an evaluation plan for assessing efficacy and ability to achieve MSP objectives, possibly measured against predetermined criteria, including procedures for regular analysis and interpretation of monitoring data to inform adaptation needs and/or for open and accessible reporting of evaluation findings. | An evaluation plan is demonstrated to assess efficacy and ability to achieve MSP objectives, measured against predetermined criteria, including procedures for regular analysis and interpretation of monitoring data to inform adaptation needs and for open and accessible reporting of evaluation findings. |
|  | Modification | No recognized need for mechanisms for modifying MSP goals, objectives, and/or management measures. | Recognized need for mechanisms for modifying MSP goals, objectives, and/or management measures based on monitoring and evaluation, MSP may be flexible enough to be modified in response to a changing ecosystem and/or governance conditions in the short- and/or long-term. | Mechanisms identified for modifying MSP goals, objectives, and/or management measures based on monitoring and evaluation. MSP and/or the spatial management plan are flexible enough to be modified in response to changing ecosystems and/or governance conditions in the short- and long-term. | Mechanisms demonstrated for modification of MSP goals, objectives, and/or management measures based on monitoring and evaluation. MSP and the spatial management plan are flexible enough to be modified in response to changing ecosystems and governance conditions in the short- and long-term. |
|  | Uncertainty | No recognized need for mechanisms to make decisions under uncertainty. | Recognized need for mechanisms to make decisions under uncertainty related to the environmental and/or socio-economic contexts of MSP, possibly including use of the precautionary approach and/or identifying missing information and/or applied research needs to reduce uncertainty for future iterations of MSP. | Mechanisms identified to make decisions under uncertainty related to the environmental and socio-economic contexts of MSP, possibly including use of the precautionary approach to overcome uncertainty and/or identifying missing information and applied research needs to reduce uncertainty for future iterations of MSP. | Mechanisms demonstrated to make decisions under uncertainty related to the environmental and socio-economic contexts of MSP, including use of the precautionary approach to overcome uncertainty and identifying missing information and applied research needs to reduce uncertainty for future iterations of MSP. |
|  | Climate change | No recognized need for mechanisms to incorporate climate change in MSP. | Recognized need for mechanisms for incorporating climate change in MSP, possibly including recognition of climate change in MSP objectives, plans, and/or policies, climate-related risks, use of climate change scenarios to anticipate changes over time and space, and/or use of dynamic management. | Mechanisms identified for incorporating climate change in MSP, including recognition of climate change in MSP objectives, plans, and/or policies, climate-related risks, use of climate change scenarios to anticipate conflicts and changes in ecosystems, ecosystem services, and human activities over time and space, and/or use of dynamic management. | Mechanisms demonstrated for incorporating climate change in MSP, including recognition of climate change in MSP objectives, plans, and policies, analysis of climate-related risks, use of climate change scenarios to anticipate conflicts and changes in ecosystems, ecosystem services, and human activities over time and space, and possibly use of dynamic management. |
|  | Biodiversity conservation | No recognized need for policies and/or management measures to maintain or restore biodiversity. | Recognized need for policies and/or management measures to maintain or restore biodiversity, including native, threatened or endangered, and/or key species, their habitats, and/or ecological processes essential to biodiversity. | Policies and/or management measures identified to maintain or restore biodiversity, including native, threatened or endangered, and/or key species, their habitats, and/or ecological processes essential to biodiversity. | Policies and/or management measures implemented to maintain or restore biodiversity, including native, threatened or endangered, and key species, their habitats, and ecological processes essential to biodiversity. |
|  | Habitat | No recognized need for policies and/or management measures to maintain or restore habitat. | Recognized need for policies and/or management measures to maintain or restore habitat quantity, quality, and/or diversity, possibly including habitat important for ecological processes, ecologically valuable species, and/or life history stages, habitat spatial arrangement, and/or relationships between habitat | Policies and/or management measures identified to maintain or restore habitat quantity, quality, and/or diversity, including habitat important for ecological processes, ecologically valuable species, and/or life history stages, habitat spatial arrangement, and/or relationships between habitats. | Policies and/or management measures implemented to maintain or restore habitat quantity, quality, and diversity, including habitat important for ecological processes, ecologically valuable species, and life history stages, habitat spatial arrangement, and relationships between habitats. |
|  | Ecosystem function | No recognized need for policies and/or management measures to maintain or restore ecological structure and function. | Recognized need for policies and/or management measures to maintain or restore ecological structure and function, possibly including biotic and abiotic ecosystem components, disturbance regimes, trophic interactions, and/or meta-population and/or community dynamics. | Policies and/or management measures identified to maintain or restore ecological structure and function, including biotic and abiotic ecosystem components, disturbance regimes, trophic interactions, and/or meta-population and community dynamics. | Policies and/or management measures implemented to maintain or restore ecological structure and function, including biotic and abiotic ecosystem components, disturbance regimes, trophic interactions, and meta-population and community dynamics. |
|  | Adjacent ecosystems | No recognized need for policies and/or management measures to improve connections between ecosystems within the planning area and to the wider land-sea environments. | Recognized need for policies and/or management measures to maintain or improve connections between ecosystems within the planning area and to the wider land-sea environments, considering geomorphology, biogeography, and/or oceanography affecting the planning area. | Policies and/or management measures identified to maintain or improve connections between ecosystems within the planning area and to the wider land-sea environments, considering geomorphology, biogeography, and/or oceanography affecting the planning area. | Policies and/or management measures implemented to maintain or improve connections between ecosystems within the planning area and to the wider land-sea environments, considering geomorphology, biogeography, and/or oceanography affecting the planning area. |
| **Ecosystem-based** | Multiple objectives | No recognized need for policies and/or management measures to balance conservation and sustainable use of biodiversity. | Recognized need for policies and/or management measures to secure the delivery of multiple ecosystem services, including tangible and/or intangible services, to balance conservation and sustainable use of biodiversity. | Policies and/or management measures identified to secure the long-term delivery of multiple ecosystem services, possibly including tangible and intangible services, to balance conservation and sustainable use of biodiversity. Ecosystem services within the marine area will be identified and will inform management priorities. | Policies and/or management measures implemented to secure the long-term delivery of multiple ecosystem services, including tangible and intangible services, to balance conservation and sustainable use of biodiversity. Ecosystem services within the marine area are identified and inform management priorities. |
|  | Minimize threats | No recognized need for policies and/or management measures to eliminate or minimize threats to species and ecosystems from human activities. | Recognized need for policies and/or management measures to eliminate or minimize threats to species and ecosystems from human activities, including identifying threats, managing ecosystems within their limits, spreading risk across the planning area, and/or addressing cumulative impacts. | Policies and/or management measures identified to eliminate or minimize threats to species and ecosystems from human activities, including identifying threats, managing ecosystems within their limits and defining those limits, spreading risk across the planning area, and/or addressing cumulative impacts. | Policies and/or management measures implemented to eliminate or minimize threats to species and ecosystems from human activities, including identifying threats, managing ecosystems within their limits, spreading risk across the planning area, and addressing cumulative impacts. |
| **Integrated** | Common framework | No recognized need to establish a common framework for integration. | Recognized need to establish a common framework that integrates within and between rightsholders, stakeholders, governance, policy, legislation, and/or management. | Clear commitment to establishing a common framework that integrates within and between rightsholders, stakeholders, governance, policy, legislation, and management. The framework may clarify how integration addresses gaps in management. | A common framework that integrates within and between rightsholders, stakeholders, governance, policy, legislation, and existing management exists. The framework clarifies how integration addresses gaps in management. |
|  | Balancing demands | No recognized need for mechanisms to ensure demands for development and protection are balanced within the planning area. | Recognized need for mechanisms to balance demands for development and protection within the planning area, possibly including evaluating trade-offs among ecological, social, cultural, and economic objectives and activities, considering cumulative impacts of multiple activities, and/or fostering economic diversification in marine sectors. | Mechanisms identified to balance demands for development and protection within the planning area, including evaluating trade-offs among ecological, social, cultural, and economic objectives and activities at temporal, spatial, and/or governance scales, considering cumulative impacts of multiple activities,  and/or fostering economic diversification in marine sectors. | Mechanisms implemented to balance demands for development and protection within the planning area, including evaluating trade-offs among ecological, social, cultural, and economic objectives and activities at temporal, spatial, and governance scales, considering cumulative impacts of multiple activities, and fostering economic diversification in marine sectors. |
|  | Multi-sector integration | No recognized need to establish an integrated management plan across sectors. | Recognized need to establish an integrated management plan across sectors within and/or affecting the planning area that may include management measures, plans, and policies and/or communicate the expectation for integration. | Clear commitment to establishing an integrated management plan across sectors within and affecting the planning area that will include management measures, plans, and policies and communicate the expectation for integration. | An integrated management plan across sectors within and affecting the planning area is demonstrated and includes their management measures, plans, and policies and communicates the expectation for integration. |
|  | Multi-level integration | No recognized need for mechanisms for implementing vertical and horizontal integration of new or existing governance and/or management systems. | Recognized need for mechanisms for implementing vertical and/or horizontal integration of new and/or existing governance and/or management systems, possibly including integration of different knowledges. | Mechanisms identified for vertical and horizontal integration of new and existing governance and/or management systems, possibly including integration of different knowledges, toward whole system management in addition to individual sub-systems. | Mechanisms implemented for vertical and horizontal integration of new and existing governance and/or management systems, including integration of different knowledges, supporting whole system management in addition to individual sub-systems. |
|  | Institutional coordination | No recognized need for mechanisms to support institutional coordination. | Recognized need for mechanisms to support institutional coordination, possibly including defining and assigning responsibility for MSP tasks and their integration, coordination of institutional programs and/or policies within and affecting the planning area, and/or commitments across institutions for implementing integrated management plans. | Mechanisms identified to support institutional coordination, including defining and assigning responsibility for MSP tasks and their integration, coordination of institutional programs and/or policies within and affecting the planning area, and/or commitments across institutions for implementing integrated management plans. | Mechanisms implemented to support institutional coordination, including defining and assigning responsibility for MSP tasks and their integration, coordination of institutional programs and policies within and affecting the planning area, and commitments across institutions for implementing integrated management plans. |
|  | Implementation | No recognized need for mechanisms for adopting and implementing integrated management plans. | Recognized need for mechanisms for adopting and implementing integrated management plans and/or resources to support implementation of integrated management plans. | Mechanisms identified for adopting and implementing integrated management plans and/or resources are identified to support the increased costs (e.g., time, money, skills) of capacity, leadership, and tools for integration. | Mechanisms demonstrated for adopting and implementing integrated management plans and resources are available to support the increased costs (e.g., time, money, skills) of capacity, leadership, and tools for integration. |
| **Participatory** | Participation plan | No recognized need to establish a participation plan. | Recognized need to establish a participation plan that may indicate who, when, and how to involve stakeholders and rightsholders, that may be developed prior to beginning MSP, and/or may define the functions and objectives of participation, MSP authorities and participants, and/or the entitlement to participate. | Clear commitment to establishing a participation plan indicating who, when, and how to involve stakeholders and rightsholders, developed prior to beginning MSP, and/or will define the functions and objectives of participation, MSP authorities and participants, and the entitlement to participate. | A participation plan is demonstrated that indicates who, when, and how to involve stakeholders and rightsholders, was developed prior to beginning MSP, and defines the functions and objectives of participation, MSP authorities and participants, and the entitlement to participate, which may evolve over time as needed. |
|  | Balanced participation | No recognition of a need for mechanisms to ensure that a final group of stakeholders and rightsholders is balanced and/or to anticipate and resolve conflicts. | Recognized need for mechanisms to ensure that the group of stakeholders and rightsholders is balanced, representing diverse interests, possibly with equal powers in advising and decision-making, and/or to anticipate and/or resolve conflicts, possibly in a transparent and equitable manner. | Mechanisms identified to ensure that the group of stakeholders and rightsholders is balanced, representing diverse interests, possibly with equal powers in advising and decision-making, and to anticipate and/or resolve conflicts in a transparent and equitable manner. | Mechanisms implemented to ensure that the group stakeholders and rightsholders is balanced, representing diverse interests, with equal powers in advising and decision-making, and to anticipate and resolve conflicts in a transparent and equitable manner. |
|  | Multiple avenues | No recognized need for mechanisms ensuring stakeholders have multiple avenues for both vertical and horizontal participation. | Recognized need for mechanisms ensuring stakeholders have multiple avenues for both vertical (e.g., formal communications, consultation) and horizontal (e.g., dialogue, negotiation) participation. | Mechanisms identified to ensure stakeholders have multiple avenues for both vertical (e.g., formal communications, consultation) and horizontal (e.g., dialogue, negotiation) participation, possibly simultaneously. | Mechanisms exist to ensure stakeholders have multiple avenues for both vertical (e.g., formal communications, consultation) and horizontal (e.g., dialogue, negotiation) participation simultaneously. |
|  | Stakeholder empowerment | No recognized need for ensuring stakeholders have the means, skills, and knowledge to participate in MSP. | Recognized need for mechanisms to ensure stakeholders have the means, skills, and knowledge to participate with a shared sense of purpose, values, and/or rules, including policies and/or protocols for promoting trust among stakeholders and in the process. | Mechanisms identified to ensure stakeholders have the means, skills, and knowledge to participate with a shared sense of purpose, values, and/or rules, including policies and/or protocols for promoting trust among stakeholders and in the process, and/or decentralizing management to the lowest level and/or enabling participation in governance. | Mechanisms implemented to ensure stakeholders have the means, skills, and knowledge to participate with a shared sense of purpose, values, and rules, including policies and/or protocols for promoting trust among stakeholders and in the process, and/or decentralizing management to the lowest level and/or enabling participation in governance. |
|  | Participation throughout | No recognized need for mechanisms to ensure participation occurs throughout MSP. | Recognized need for mechanisms to ensure participation occurs throughout MSP, including regular meetings defined for stakeholder involvement and/or opportunity to engage in aspects of decision-making, setting objectives, developing alternatives, and/or identifying a preferred spatial management plan. | Mechanisms identified to ensure participation occurs throughout MSP, including regular meetings defined for stakeholder involvement at each MSP stage and opportunity to engage in aspects of decision-making, setting objectives, developing alternatives, and/or identifying a preferred spatial management plan. | Mechanisms implemented to ensure participation occurs throughout MSP, including regular meetings defined for stakeholder involvement at each MSP stage and opportunity to engage in each aspect of decision-making, setting objectives, developing alternatives, and identifying a preferred spatial management plan. |
|  | Engagement & communication | No recognized need for mechanisms to effectively engage and communicate with participants. | Recognized need for mechanisms to effectively engage and communicate with participants, including timely reporting and frequent contact, targeted and/or accessible communication, communication of evaluation and adaptation, designation of a lead communicator, and/or identification of resources. | Mechanisms identified to effectively engage and communicate with participants, including timely reporting and frequent contact, targeted and accessible communication, communication of evaluation and adaptation processes, designation of a lead communicator, and/or identification of resources. | Mechanisms implemented to effectively engage and communicate with participants, including timely reporting and frequent contact, targeted and accessible communication in multiple formats, communication of evaluation and adaptation processes, designation of a lead communicator, and identification of resources. |
|  | Spatial management plan | No recognized need to develop a preferred spatial management plan. | Recognized need to develop a spatial management plan, identifying when, where, and how the goals and objectives of MSP will be met, that may be formally adopted. The plan may identify boundaries, funding, institutional arrangements, rules, incentives and disincentives, and/or management measures. | Clear commitment to developing a preferred spatial management plan, possibly from alternative scenarios, identifying when, where, and how the goals and objectives of MSP will be met, and possibly adopted through a formal process. The plan will identify boundaries, funding, institutional arrangements, rules, incentives and disincentives, and/or management measures. | A preferred spatial management plan is demonstrated, developed from alternative scenarios, identifying when, where, and how the goals and objectives of MSP will be met, and is adopted through a formal process. The plan identifies boundaries, funding, institutional arrangements, rules, incentives and disincentives, and management measures. |
| **Place-based** | Boundaries | No recognized need to establish boundaries. | Recognized need to establish boundaries for the planning area, possibly prior to initiating MSP, including geographical, administrative, and/or analytical boundaries at local, national, and transnational scales as needed. | Clear commitment to establishing boundaries for the planning area prior to initiating MSP, including geographical, administrative, and/or analytical boundaries at local, national, and transnational scales as needed. | Boundaries are demonstrated for the planning area prior to initiating MSP, including geographical, administrative, and analytical, boundaries at local, national, and transnational scales as needed. |
|  | Upstream & downstream | No recognition of upstream (i.e., affecting the planning area) and/or downstream (i.e., affected by the planning area) human activities and pressures. | MSP generally recognizes upstream (i.e., affecting the planning area) and/or downstream (i.e., affected by the planning area) human activities and pressures. | MSP identifies specific upstream (i.e., affecting the planning area) and/or downstream (i.e., affected by the planning area) human activities and pressures. | MSP demonstrably incorporates specific upstream (i.e., affecting the planning area) and downstream (i.e., affected by the planning area) human activities and pressures in the spatial management plan. |
|  | Spatial information | No recognized need to develop an inventory of spatial data. | Recognized need to develop an inventory of spatial data, including current and/or future trends in ecological, oceanographic, and human activity data derived from multiple sources. | Clear commitment to developing an inventory of spatial data, including current and future trends in ecological, oceanographic, and human activity data derived from scientific literature, expert opinion, government sources, local and/or traditional knowledge, and/or direct measurements. | An inventory of spatial data is demonstrated, including current and future trends in ecological, oceanographic, and human activity data derived from scientific literature, expert opinion, government sources, local and traditional knowledge, and/or direct measurements. |
|  | Scale | No recognized need to ensure MSP occurs at sufficient temporal and spatial scales. | Recognized need to ensure that MSP occurs at temporal and spatial scales sufficient to capture interactions between social, ecological, and/or oceanographic components of the planning area to support long-term objectives and/or goals. | Clear commitment to ensuring that MSP occurs at temporal and spatial scales sufficient to capture interactions between social, ecological, and oceanographic components of the planning area to support long-term objectives and/or goals. | MSP is demonstrated to occur at temporal and spatial scales sufficient to capture interactions between social, ecological, and oceanographic components of the planning area to support long-term objectives and goals. |
|  | Conflicts & compatibilities | No recognized need to analyze spatial and temporal distributions and density of human activities to assess conflicts and compatibilities. | Recognized need to analyze spatial and temporal distributions and density of human activities to assess conflicts and compatibilities among existing uses, existing uses and the environment, and/or existing and future uses to inform the spatial management plan. | Clear commitment to analyzing spatial and temporal distributions and density of human activities to assess conflicts and compatibilities among existing uses, existing uses and the environment, and existing and future uses, possibly including a priori allocation of areas to future uses, to inform the spatial management plan. | Spatial and temporal distributions and density of human activities are demonstrably analyzed to assess conflicts and compatibilities among existing uses, existing uses and the environment, and existing and future uses, possibly including a priori allocation of areas to future uses, to inform the spatial management plan. |
| **Strategic** | Resources | No recognized need for mechanisms to ensure sustainable human, technical, and/or financial resources are available to develop and implement the current and/or future iterations of MSP. | Recognized need for mechanisms to ensure sustainable human, technical, and/or financial resources are available to develop and implement the current and/or future iterations of MSP. | Mechanisms identified to ensure sustainable human, technical, and/or financial resources are available to develop and implement the current and/or future iterations of MSP over the long-term, possibly including a financial plan for MSP costs, means to multiple and alternative financial resources, and/or identification of appropriately skilled staff. | Mechanisms implemented to ensure sustainable human, technical, and financial resources are available to develop and implement the current and future iterations of MSP over the long-term, including a financial plan for MSP costs, means to multiple and alternative financial resources, and identification of appropriately skilled staff. |
|  | Vision, goals & objectives | No recognized need to establish goals, objectives, and guiding principles at the onset of MSP. | Recognized need to establish a vision, goals, SMART objectives, and guiding principles for MSP at the onset of MSP that are supported by decision-makers and stakeholders. | A clear commitment to establishing a vision, goals, SMART objectives, and guiding principles for MSP at the onset of MSP that are supported by decision-makers and stakeholders. | A vision, goals, SMART objectives, and enforceable guiding principles for MSP are demonstrated, established at the onset of MSP and supported by decision-makers and stakeholders. This may include a list of specific problems to be solved by MSP. |
|  | Authority & leadership | No recognized need to establish authorities or MSP team to lead MSP. | Recognized need to establish authorities, political champions, a coordinating agency, and/or MSP team to lead MSP and/or the implementation of the spatial management plan. | Clear commitment to establishing authorities, political champions, a coordinating agency, and/or MSP team to lead MSP and the implementation of the spatial management plan, possibly prior to beginning MSP. | Authorities, political champions, a coordinating agency, and MSP team are established to lead MSP and the implementation of the spatial management plan, possibly prior to beginning MSP. |
|  | Work plan | No recognized need to establish a work plan. | Recognized need to establish a work plan that identifies key work products and their interdependencies, assigns responsibility for outputs, identifies resources required to deliver outputs, sets a timeframe for initiating, completing, and revising MSP, and/or identifies potential risks in MSP and contingency measures. | Clear commitment to establishing a work plan that identifies key work products and their interdependencies, assigns responsibility for outputs, identifies resources for outputs, sets an iterative timeframe for initiating, completing, and revising MSP, and/or identifies potential risks and contingency measures or a work plan exists containing some requirements. | A work plan is demonstrated that identifies key work products and their interdependencies, assigns responsibility for outputs, identifies resources required to deliver outputs, sets an iterative timeframe for initiating, completing, and revising MSP, and identifies potential risks in MSP and contingency measures. |
|  | Evidence-based | No clear intention to be evidence-based. | Clear intention to be evidence-based, using the best available natural and/or social science and/or different types of information relevant to the planning area. | Clear commitment to being evidence-based, possibly informed by a science advisory body or similar, using the best available natural and social science and/or different types of information relevant to the planning area and/or external environment. | MSP is demonstrably evidence-based, informed by a science advisory body or similar, using the best available natural and social science and different types of information (e.g., scientific, Indigenous and local knowledge, and knowledge innovations and practices) relevant to the planning area and external environment. |
|  | Compliance & enforcement | No recognized need to establish a plan and/or strategies for enforcement and compliance. | Recognized need to establish a plan and/or strategies that define authority and measures for enforcing the spatial management plan and achieving high compliance. Compliance may be defined, including which activities are subject to requirements of the spatial management plan and/or responses to non-compliance. | Clear commitment to establishing a plan and/or strategies that define authority and measures for enforcing the spatial management plan and achieving high compliance. Compliance is clearly defined, including which activities are subject to requirements of the spatial management plan and responses to non-compliance. | A plan and/or strategies are demonstrated that define authority and measures for enforcing the spatial management plan and achieving high compliance. Compliance is clearly defined, including which activities are subject to requirements of the spatial management plan and responses to non-compliance. |
